# Evaluation of Functional T-Cell Assays That Predict Causal Allergens in Eosinophilic Esophagitis

**DOI:** 10.3390/diagnostics16020175

**Published:** 2026-01-06

**Authors:** Julianna Dilollo, Cleandre M. Guerrier, Ignacio De La Torre Saenz Rico, Elizabeth Martin, Susan Lee, Michael Pratt, Pavithra Vinnakota, Walter Faig, Michele E. Paessler, Jonathan M. Spergel, David A. Hill

**Affiliations:** 1 Division of Allergy and Immunology, Children’s Hospital of Philadelphia, Philadelphia, PA 19104, USApv3319@pcom.edu (P.V.);; 2 Westat, 3535 Market St #1035, Philadelphia, PA 19104, USA; 3 Department of Pathology and Laboratory Medicine, Children’s Hospital of Philadelphia, Philadelphia, PA 19104, USA; 4 Department of Pediatrics and Institute for Immunology and Immune Health, Perelman School of Medicine at the University of Pennsylvania, Philadelphia, PA 19104, USA

**Keywords:** food allergy, eosinophilic esophagitis, T cell, blood test, assay

## Abstract

**Background**: Eosinophilic esophagitis (EoE) is a chronic, food antigen-driven disease of the esophagus that causes considerable morbidity. Elimination of allergenic foods from a patient’s diet is a highly effective treatment. However, existing allergen testing modalities are not effective at identifying EoE-causal foods. We sought to determine the extent to which positive results for two functional T-cell assays, the EoE Milk Test and EoE Soy Test, associated with the clinical outcomes of EoE milk allergy and EoE soy allergy, respectively. **Methods**: Subjects were enrolled into one of two study designs: a prospective observational study or a retrospective case/control study. Additional control samples were obtained from an institutional core. The EoE Milk and Soy Tests were performed on peripheral blood samples, and the association between positive tests and clinical outcomes was determined using Receiver Operating Characteristic curves and other performance measures. **Results**: The EoE Milk Test maintained reliability regardless of disease activity or recent milk consumption and had 87% sensitivity and 83% specificity for EoE milk allergy in all study subjects (control and EoE). The EoE Soy Test had 90% sensitivity and 93% specificity in all subjects. **Conclusions**: Our evaluation of the EoE Milk and Soy Tests demonstrates that these functional T-cell assays hold promise as a predictive tool for identifying causal allergens in eosinophilic esophagitis patients.

## 1. Introduction

Eosinophilic esophagitis (EoE) is a chronic, food antigen-driven disease of the esophagus that affects up to 1 in 700 people and has an incidence that is increasing by 12–17% per year [1,2,3,4]. Most EoE patients have a single causal food [5], and milk is the most common inciting allergen [5,6,7]. EoE symptoms and sequelae include chest and abdominal pain, difficulty swallowing, malnutrition, esophageal stricture formation, and food impaction [8]. In addition to primary gastrointestinal manifestations, EoE also predisposes to secondary conditions such as anxiety [9], sleep disturbance [10], and eating disorders [11]. As a result, EoE has a significant negative effect on the quality of life of both patients and their caregivers [12,13].

It is imperative to control EoE inflammation to minimize symptoms and avoid long-term, life-altering sequelae such as stricture formation [2,14,15]. Due to uncertainties and ineffectiveness of some medical therapies [16,17], a preferred therapeutic strategy for many patients and caregivers is identification and removal of causal food(s) from a patient’s diet [18,19,20]. However, studies of existing allergen testing have revealed less than a 50% success rate in identifying EoE-causal foods [21]. Further, experimental attempts at utilizing food allergen-specific IgG4 levels to predict EoE-causal foods have had mixed results [22], particularly in children [23]. As a result, it is generally accepted that existing allergen testing modalities are not helpful for clinical management [21,24,25].

In the absence of effective clinical testing, clinicians perform EoE-causal food identification via cycles of empiric food elimination, followed by endoscopy and esophageal biopsy [26]. Initial studies on six-food empiric elimination diets targeted the most common EoE-causing foods [27]. More recent randomized trials compared one- versus six-food elimination in adults [28] and one- versus four-food elimination in children [29], both showing similar efficacy—approximately 40% of patients achieved symptomologic and clinical resolution with identification of the causative food. A four-food elimination with sequential reintroduction yielded slightly better outcomes, identifying all EoE-causal foods in 64% of subjects over 32 weeks [5]. Despite these advances, the current standard of care remains invasive and inefficient, resulting in unnecessary procedures and prolonging the time until disease remission.

These realities underscore a critical need for the development of new clinical tools to aid in the identification of EoE-causal foods. A key step in this development is determining whether circulating, food-activated memory T-cell responses can serve as surrogates for a patient’s clinically defined EoE allergen profile. To address this, we developed minimally invasive methods to detect and quantify food antigen-activated memory T cells in EoE patients, collectively known as EoE Food Tests [23,30]. In a retrospective study, the EoE Milk Test demonstrated high sensitivity (88%) and specificity (82%) in correlating with EoE milk allergy [23]. Here, we used prospective observational and retrospective case/control study designs to assess the EoE Milk Test’s predictive value in real-world clinical practice, establish preliminary quality control data, and determine initial performance characteristics for a novel EoE Soy Test.

## 2. Materials and Methods

Study design and oversight: This project utilized two study designs: a prospective observational study design and a retrospective case/control study design. The study was conducted at the Children’s Hospital of Philadelphia (CHOP). During the prospective observational study, subjects with symptoms of EoE were enrolled at the time of endoscopy and a 3–15 mL blood sample was obtained. Functional T-cell assays were performed and analyzed prior to the release of endoscopy results. Subjects were tracked via periodic review of their electronic medical record (EMR; Epic, Verona, WI, USA) for the outcome of EoE diagnosis and the clinical determination of the contribution of either milk and/or soy to their EoE presentation. Chart review began one week after enrollment and continued quarterly for the EoE group until allergy or tolerance to milk and/or soy was determined, or until the study was concluded.

During the retrospective case/control study, subjects with clinically determined EoE soy allergy OR established tolerance to soy were identified via EMR review and enrolled at their routine appointment. A 10–15 mL blood sample was obtained and the EoE Soy Test was performed. Subjects’ clinical course was subsequently monitored via EMR review, and data were collected and analyzed by the investigators. Written informed consent and assent (when applicable) was obtained from all the patients or their parent or legal guardian before enrollment. Additional deidentified control samples with limited associated clinical information were obtained from the Human Immunology Core at the University of Pennsylvania for use in repeatability experiments, under their IRB protocol.

Subjects, enrollment criteria, and endpoints: CHOP patients eligible for the prospective study were tetanus vaccinated, had symptoms of EoE, and were undergoing a clinically indicated endoscopy. CHOP patients eligible for the retrospective study were tetanus vaccinated, and either had EoE, as established by a previous endoscopic biopsy and consistent with international diagnostic guidelines (peak eosinophil count, ≥15 eosinophils per high-power field (eos/hpf)), or did not have EoE [31]. Other causes of esophageal eosinophilia were ruled out by the treating physician [31]. For both studies, an EoE-causal food (allergy) to milk and/or soy was defined by the following criteria: addition of a single food led to exacerbation of esophageal eosinophilia (increase of greater than 15 eos/hpf), or removal of a single food led to normalization of biopsy (esophageal eosinophilia showed less than 15 eos/hpf). Subjects treated with an immunomodulatory medication (e.g., swallowed steroids, dupilumab) or with high dose PPI in combination with diet changes were not assigned a milk or soy status, as remission while on these therapies cannot be attributed to the removal of an EoE-causal food. Subjects under combination treatment were followed and assigned a status only if medication was ceased or PPI dosage reduced while diet adherence continued for at least three months. Endpoints for the observational trial were as follows: (1) absence of EoE (control subject), (2) presence of EoE with a clinically determined contribution of milk and/or soy, (3) presence of EoE with unknown contribution of milk and/or soy. Samples from the Human Immunology Core at the University of Pennsylvania were not assigned an outcome.

Sample isolation and culture: Peripheral blood samples were obtained and peripheral blood mononuclear cells (PBMCs) were isolated, carboxyfluorescein succinimidyl ester (CFSE) labeled (Invitrogen, Waltham, MA, USA; REF C34554), and cultured as previously described [23]. PBMCs were cultured for six days in the absence or presence of endotoxin-depleted milk (6.25 μg of each of α-lactalbumin, β-lactoglobulin, α-casein, β-casein, and κ-casein, Millipore Sigma, Burlington, MA, USA), soy (5.0 μg, Greer Labs, Lenoir, NC, USA, F209), or tetanus toxoid (TT, 0.625 μg, Astarte Biologics, Memphis, TN, USA) proteins, per 200,000 cells. PBMCs from the Human Immunology Core were isolated by apheresis and delivered in RPMI-1640.

Flow cytometric evaluation: PBMCs were harvested and treated with Live/DEAD Fixable Blue (Invitrogen) for 15 min at 4 °C in the dark. Washed PBMCs were stained with anti-human CD8 (SK1, BioLegend, San Diego, CA, USA, 4:100, BV510), CD3 (SK7, BD Biosciences, Franklin Lakes, NJ, USA, 5:100, BUV395), CD4 (OKT4, BioLegend, 4:100, BV605), CD19 (HIB19, BioLegend, 4:100, AF700 or BV711), CD45RA (HI100, BD Biosciences, 4:100, V450, or BioLegend, 4:100, BV786), and CD45RO (UCHL1, BioLegend, 5:100, BV650) for 30 min at 4 °C in the dark. Cells were washed, fixed (Invitrogen), and stored at 4 °C. Data were acquired within 24 h of harvest on a core maintained LSR Fortessa (BD). The cytometer was compensated using OneComp eBeads (Invitrogen), unstained cells, CFSE stained cells, and Live/DEAD Fixable Blue stained dead cells. 250,000–1,000,000 events were acquired for each culture condition. Data were analyzed using FlowJo software (version 10.10, BD). Memory CD4+ T cells were gated as previously described [23]. Proliferation of memory CD4+ T cells in unstimulated, tetanus toxoid-stimulated, and milk or soy protein-stimulated arms was measured by CFSE dilution.

Data curation: Some subject samples were excluded during the study due to insufficient blood sample (<3 mL), technical exclusions (e.g., high background proliferation, poor tetanus response), or clinical exclusions (e.g., therapy course that did not include dietary food elimination, poor diet adherence, medication use which obfuscated the impact of diet changes). Proliferation of 20% or higher in the unstimulated condition was defined as failure of the negative control. A response to tetanus toxoid of 2% greater than background was chosen as the minimum threshold for a successful positive control, consistent with the previously published threshold [23].

Two variables negatively impacting the performance of the EoE Milk Test were identified during the analysis of the observational study. These included data from assays with fewer than 1 million cells per experimental arm at the start of culture and those with viability <70%. These samples were excluded from the final analyses.

By Fisher’s exact tests, the original milk-tolerant control cohort of >50 subjects were significantly older, more racially and ethnically diverse, and less atopic than the milk-allergic group. We performed a statistically guided pruning of the observational study control group to match the demographic characteristics more closely to those of the EoE milk-allergic group (considered clinical exclusions, see statistical analyses). Assay performance characteristics were evaluated using Receiver Operating Characteristic (ROC) curves, as well as other performance measures, such as positive predictive value (PPV), negative predictive value (NPV), Youden index, and accuracy.

Statistical analysis: Summary statistics by allergen or tolerance status are presented for the combined cohort and stratified by EoE and control subjects. Age at relevant assay is reported as the mean, while all other variables are shown as percentages. Pruning of the milk tolerant control cohort was based on a 2:1 match to EoE-milk subjects based on patient race (White vs. non-White) and age categorization (≤10 years vs. >10) from the randomized control sample. Demographic differences between milk-allergic and milk-tolerant individuals were analyzed using Fisher’s exact tests, and the final cohorts did not differ across demographics other than age. The soy-tolerant and soy-allergic cohorts did not differ across any demographic categories by Fisher’s exact tests. EoE Milk and Soy Test performance was assessed using ROC curves, with the %P1 (milk or soy/tetanus) cutoff determined by the optimal operating point (closest to perfect performance). Sensitivity, specificity, PPV, NPV, AUC, Youden’s index, and accuracy are reported for each ROC curve. Differences in mean %P1 (milk or soy/tetanus) were evaluated using Mann–Whitney U tests. These analyses were conducted for the full cohort and for EoE subjects alone, with controls limited to non-milk EoE cases.

The association of milk test outcomes (background proliferation, milk proliferation, and milk/tetanus proliferation) with disease activity (Eosinophils/HPF) is given as linear regression results including R^2^ and significance of milk test outcome estimates. Differences between those consuming or avoiding milk were evaluated with Mann–Whitney U tests for milk proliferation as well as milk/tetanus proliferation. Background proliferation differences were assessed with Fisher’s exact test using counts below or above %20 %P1 (unstimulated).

The association of tetanus proliferation by subject age at relevant assay and by time from last vaccination was also assessed with linear regression. True positive (TP), true negative (TN), false positive (FP), false negative (FN), and unknown status individuals were given by color coding on the plots. Differences between FP and accurate predictions (TP + TN) were compared by Mann–Whitney U tests.

Standard deviation and coefficient of variation are given for milk test outcomes for the repeatability cohort. This cohort included five prospectively recruited pediatric subjects (one with EoE milk allergy, two with EoE caused by unknown foods, and two without EoE) and seven adult subjects enrolled on the Human Immunology Core’s IRB protocol. Paired t-tests were used to determine differences across repeat assay outputs. Repeat assays were performed in parallel by splitting the blood (prospective) or PBMC (Human Immunology Core) sample in two prior to CFSE staining and subsequent assay procedures. All inferential statistics used two-sided tests and significance level 0.05. All figures and analyses were performed using Prism (GraphPad, version 10.6.1).

## 3. Results

**Figure 1:**
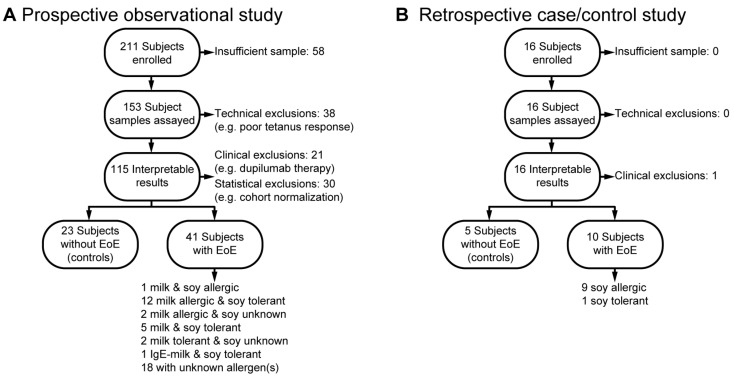
Enrollment in the prospective and retrospective studies. (**A**) Prospective observational study enrollment. (**B**) Retrospective case/control study enrollment.

To evaluate the predictive accuracy of the EoE Milk and Soy Tests, we used two study designs. In a prospective observational study, we enrolled 211 subjects with EoE symptoms at initial endoscopy. Blood samples were tested using the EoE Milk and/or Soy Tests, and electronic medical records were reviewed to confirm clinical diagnoses of EoE-related milk and/or soy allergy (Figure 1A). During the study, we identified the following groups: 1 subject allergic to both milk and soy, 12 subjects with a milk allergy and soy tolerance, and 2 subjects with a milk allergy and unknown soy status. Additionally, five subjects were tolerant to both milk and soy, two subjects tolerated milk but had an unknown soy status, and one subject had an IgE-mediated milk allergy but was tolerant to soy. Participants with confirmed clinical tolerance to milk or soy were combined with non-EoE control samples in the subsequent analyses of the test performance characteristics.

During our observational study, we noted that insufficient numbers of clinically confirmed EoE soy allergy were being identified. To address the limited number of subjects, we implemented a supplemental retrospective case/control study design (Figure 1B). In this design, we reviewed the EMR to identify and enroll 16 subjects with or without confirmed EoE soy allergy. This retrospective analysis identified nine subjects with EoE soy allergy, one subject with EoE who was known to be soy tolerant, and five non-EoE controls. As both studies were designed to be of minimal risk to the subjects, we had several subject samples that were not of sufficient quantity to be assayed. Technical exclusions included high background T-cell proliferation and poor tetanus response. Clinical exclusions included adoption of a therapy plan that did not allow for the assessment of the contribution of milk and/or soy to the subject’s EoE (e.g., dupilumab). A summary of final study cohort demographic and clinical characteristics, both overall and by subject, can be found in Table 1, Table A1, Table A2, Table A3 and Table A4. There were not significant differences between milk-allergic and milk-tolerant individuals regarding sex, race, or ethnicity.

**Figure 2:**
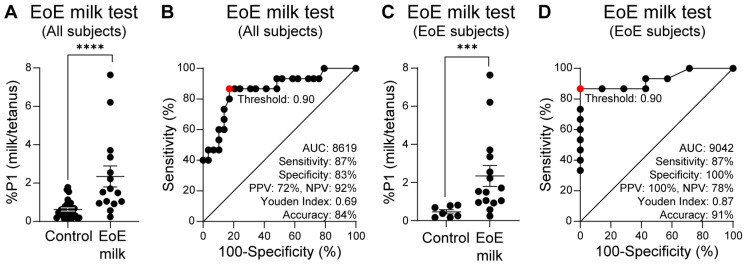
Prospective study of the EoE Milk Test. (**A**) Ratio of milk vs. tetanus expanded cells (%P1) from milk tolerant controls with or without EoE (*N* = 29), and EoE milk subjects (*N* = 15). (**B**) ROC curve of panel A data. (**C**) Ratio of milk vs. tetanus expanded cells (%P1) from milk-tolerant control subjects with EoE (*N* = 7) and EoE milk subjects (*N* = 15). (**D**). ROC curve of panel (**C**) data. Mean ± SEM shown. *p*-value = 0.0008 (***); ≤0.0001 (****) by Mann–Whitney U test. Statistics shown for optimal operating point of 0.90, indicated in red.

As previously described [23], we used the percentage of divided CD4+ CD45RO+ memory T cells in a subject’s food protein-stimulated PBMC culture arm, normalized to the percentage of divided memory T cells in a subject’s tetanus-stimulated PBMC culture arm, (%P1 food/tetanus) as the assay readout. In the observational study, subjects who were subsequently diagnosed with EoE milk allergy had a significantly higher proportion of memory T cells that divided upon milk stimulation, as compared with control subjects (Figure 2A). To quantify the extent to which a positive EoE Milk Test prospectively associated with clinical EoE milk allergy, we employed an ROC curve, as well as other performance measures. Based on this ROC curve, we defined a “positive” EoE Milk Test outcome as a %P1 milk/tetanus ratio of ≥0.90. In this analysis, we found that a positive EoE Milk Test was 87% sensitive and 83% specific for EoE milk allergy in all study subjects (both control and EoE milk allergy) (Figure 2B). This equated to a PPV of 72%, a NPV of 92%, a Youden index of 0.69, and an accuracy of 84%.

The most relevant use case for the EoE Food Tests is after EoE diagnosis and before implementation of a personalized food elimination diet. To evaluate the performance characteristics of the EoE Milk Test in this specific use case, we repeated our performance analysis in EoE subjects only. For this sub-analysis, the control group was subjects with EoE who were found to tolerate milk. In this sub-analysis, we again observed that subjects who were subsequently diagnosed with EoE milk allergy had a significantly higher proportion of memory T cells that divided upon milk stimulation, as compared with milk-tolerant EoE subjects (Figure 2C). Further, again using a “positive” EoE Milk Test outcome as a %P1 milk/tetanus ratio of ≥0.90, we observed that the EoE Milk Test performance characteristics improved to a sensitivity of 87%, specificity of 100%, PPV of 100%, NPV of 78%, Youden index of 0.87, and accuracy of 91% (Figure 2D).

**Figure 3:**
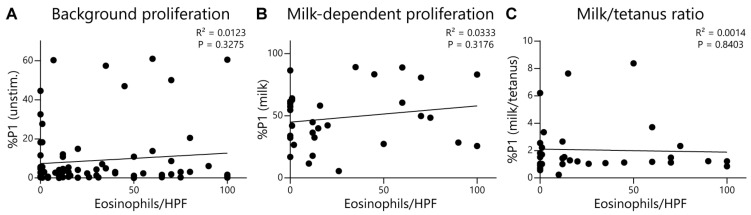
Lack of association between key EoE Milk Test assay readouts and disease activity. (**A**) Association between the %P1 cells in unstimulated (unstim.) PBMC cultures and eosinophils per high-power field (HPF) of EoE subjects. Data represent 80 assays from *N* = 69 individuals with EoE. (**B**) Association between the %P1 cells in milk-stimulated PBMC cultures and eosinophils per HPF of subjects with EoE milk allergy. Data represent 32 assays from *N* = 25 individuals with EoE milk allergy. (**C**) Association between the ratio of the %P1 cells in milk-stimulated vs. tetanus-stimulated PBMC cultures of subjects with EoE milk allergy. Data represent 32 assays from *N* = 25 individuals with EoE milk allergy. R^2^ and *p* values determined by simple linear regression.

**Figure 4:**
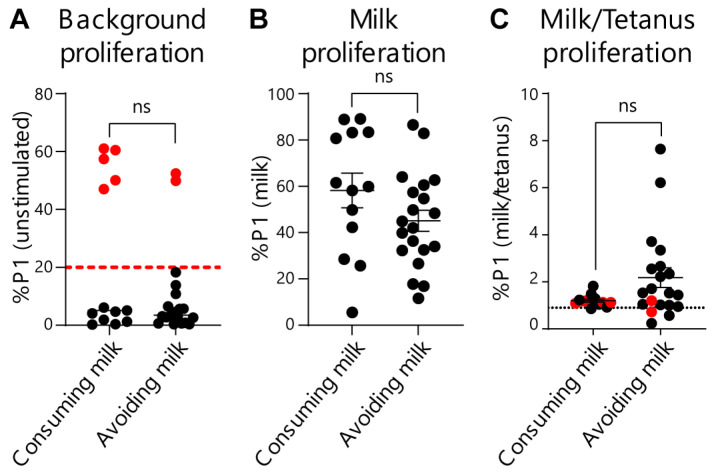
Effects of milk consumption on key EoE Milk Test outcomes. (**A**) %P1 cell population in unstimulated cultures of PBMCs from subjects with EoE milk allergy who were consuming or avoiding milk. Dashed red line indicates cutoff for “high background” exclusion criteria. *p*-value not significant (ns) as determined by Fisher’s exact test. (**B**) %P1 cell population in milk-stimulated cultures of PBMCs from subjects with EoE milk allergy who were consuming or avoiding milk. Mean ± SEM shown. *p*-value not significant (ns) as determined by Mann–Whitney U test. (**C**) Ratio of the %P1 cell population in milk-stimulated vs. tetanus-stimulated PBMC cultures from subjects with EoE milk allergy who were consuming or avoiding milk. *N* = 11 (consuming) and 16 (avoiding). Dotted black line indicates the threshold for assay “positivity”. Red dots indicate high-background assays that were excluded from Figure 2. Mean ± SEM shown. *p*-value not significant (ns) as determined by Mann–Whitney U test. Data represent 33 assays from *N* = 24 individuals with EoE milk allergy.

To further evaluate and provide rigor to our EoE Milk Test, we performed several additional analyses. We first assessed whether the degree of disease activity (as measured by eosinophils per high powered field) at the time of testing influenced test outcomes. We did not observe a significant relationship between background T-cell proliferation (proliferation in the absence of a stimulus) (Figure 3A), milk-dependent proliferation (Figure 3B), or the %P1 milk/tetanus ratio (Figure 3C), and disease activity. Second, we evaluated if milk consumption at the time of testing influenced the outcome of the EoE Milk Test. However, background T-cell proliferation (Figure 4A), milk-dependent T-cell proliferation (Figure 4B), and the %P1 milk/tetanus ratio (Figure 4C) were similar between subjects who were consuming or avoiding milk at the time of assay.

**Figure 5:**
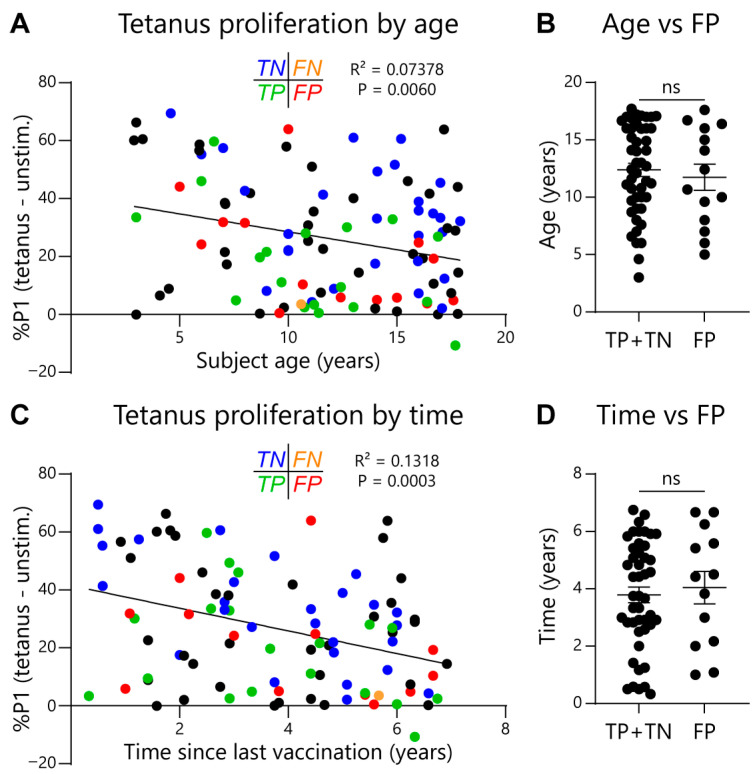
Association between subject age or time since last vaccination on tetanus toxoid-dependent T-cell proliferation. (**A**) Association between the magnitude of the tetanus response (%P1 cells in tetanus-stimulated minus unstimulated PBMC cultures) and subject age in tetanus toxoid-vaccinated subjects. Test concordance with clinical outcome indicated as follows: true negative (TN, blue); false negative (FN, orange); true positive (TP, green); false positive (FP, red); black = no clinical determination of EoE causal allergens. R^2^ and *p* values determined by simple linear regression. (**B**) Statistical evaluation between FP test outcome and age. *N* = 46 (TP + TN) and 14 (FP). Mean ± SEM shown. *p*-value not significant (ns) as determined by Mann–Whitney U test. (**C**) Association between the magnitude of the tetanus response and the length of time since the last tetanus-toxoid vaccination. R^2^ and *p* values determined by simple linear regression. (**D**) Statistical evaluation between FP test outcome and the length of time since the last tetanus-toxoid vaccination. *N* = 46 (TP + TN) and 14 (FP). Mean ± SEM shown. *p*-value not significant (ns) as determined by Mann–Whitney U test.

**Figure 6:**
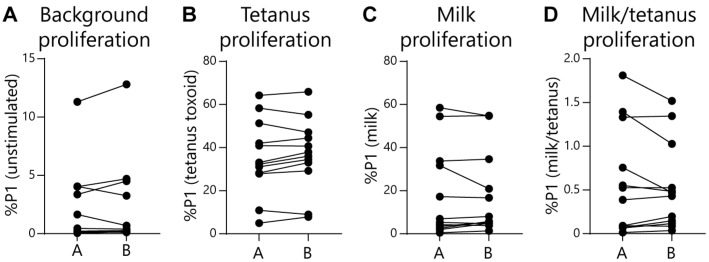
Repeatability of the EoE Milk Test. (**A**) Independent measurements (A vs. B) of the %P1 cells in unstimulated cultures. Standard deviation (SD) = 0.33, coefficient of variation (CV) = 0.23, *N* = 12. (**B**) Independent measurements (A vs. B) of %P1 cells in tetanus toxoid-stimulated cultures. SD = 2.0, CV = 5.0, *N* = 12. (**C**) Independent measurements (A vs. B) of %P1 cells in milk-stimulated cultures. SD = 1.6, CV = 6.6, *N* = 12. (**D**) Independent measurements (A vs. B) of the ratio of %P1 cells in milk-stimulated as compared to tetanus-stimulated cultures. SD = 0.08, CV = 0.01, *N* = 12. No plots displayed significant differences as determined by paired *t*-tests.

Given the EoE Food Tests rely on normalizing food-activated T-cell responses to a subject’s tetanus-activated T-cell response, it could be possible for false-positive test rates to increase as tetanus responses wane. To check for this effect, we compared subject age or time since last tetanus vaccination with assay outcomes. As expected, we observed that both subject age (Figure 5A) and time since last tetanus vaccination (Figure 5C) negatively correlated with the magnitude of the tetanus-activated T-cell response. However, the false-positive rate for the EoE Milk Test did not significantly correlate with either subject age or time since last tetanus vaccination (Figure 5B,D). In addition, we assessed repeatability of the EoE Milk Test by performing duplicate assays in a subset of subjects. We did not observe a significant difference in background proliferation, tetanus-dependent proliferation, milk-dependent proliferation, or the %P1 milk/tetanus ratio between parallel tests (Figure 6).

**Figure 7:**
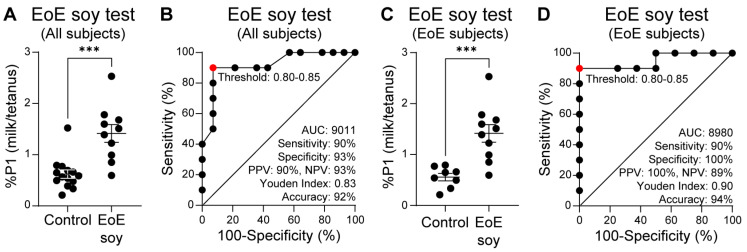
EoE Soy Test. (**A**) Ratio of soy vs. tetanus expanded cells (%P1) from soy-tolerant control subjects with or without EoE (*N* = 14), and EoE soy subjects (*N* = 10). (**B**) ROC curve of panel A data. (**C**) Ratio of soy vs. tetanus expanded cells (%P1) from soy-tolerant control subjects with EoE (*N* = 8), and EoE soy subjects (*N* = 10). (**D**) ROC curve of panel C data. Mean ± SEM shown. *p*-value ≤ 0.0005 (***) by Mann–Whitney U test. Statistics shown for optimal operating point of 0.80-0.85, indicated in red.

Finally, we sought to evaluate the performance characteristics of our EoE Soy Test. Because of the limited number of subjects with clinically confirmed EoE soy allergy in our observational study, we supplemented EoE soy subject data with additional, retrospectively recruited subjects. In this combined analysis, we observed that subjects with EoE soy allergy had a significantly higher proportion of memory T cells that divided upon soy stimulation, as compared with control subjects (Figure 7A). Based on the ROC curve for these data, we defined a “positive” EoE Soy Test outcome as a %P1 milk/tetanus ratio of ≥0.80. In all subjects, the EoE Soy Test performance characteristics were a sensitivity of 90%, specificity of 93%, PPV of 90%, NPV of 93%, Youden index of 0.83, and accuracy of 92% (Figure 7B). In EoE subjects only (Figure 7C), the EoE Soy Test had a sensitivity of 90%, specificity of 100%, PPV of 100%, NPV of 89%, Youden index of 0.90, and accuracy of 94% (Figure 7D).

## 4. Discussion

Our evaluation of the EoE Milk and Soy Tests demonstrates that these functional T-cell assays hold promise as a predictive tool for identifying causal allergens in eosinophilic esophagitis. By integrating a minimally invasive blood test into clinical workflows, this approach has the potential to reduce reliance on serial dietary elimination and endoscopic biopsy, thereby expediting the time to symptom resolution and disease control, minimizing the risk of chronic EoE sequela, and reducing the total number of dietary changes and endoscopies.

We previously employed a retrospective, case/cohort design to establish feasibility and clinical association of the EoE Milk Test [23]. However, retrospective studies have inherent limitations, including those related to the temporal sequence of events. In contrast, a prospective design ensures that the exposure (in this case the diagnostic test) precedes the outcome, which is critical for establishing causality and assessing the true performance of a diagnostic test over time. In our prospective observational study, the assay accurately identified patients with EoE milk allergy, aligning with our findings from a prior study [23]. While the EoE Soy Test could not be evaluated prospectively and our findings are thus limited by the retrospective study design, the EoE Soy Test performance compared equitably to the retrospective performance of the EoE Milk Test [22]. These findings reinforce the notion that circulating, food-activated T-cell responses serve as a reliable biomarker for food antigen-driven esophageal inflammation.

A key advantage of the EoE Food Tests is their ability to provide individualized predictions of food tolerance and allergy, which may facilitate more targeted dietary interventions. This contrasts with the current empirical approach of elimination diets, which often necessitate prolonged periods of dietary restriction and multiple endoscopies to confirm efficacy [28,29]. Our data suggest that integrating the EoE Food Tests into clinical decision making could streamline patient management, minimizing unnecessary food eliminations and optimizing nutritional outcomes.

Notably, the EoE Food Tests perform better when used in patients with biopsy-confirmed EoE. As such, this patient group represents the best initial use case for the EoE Food Tests. However, additional uses cases for the EoE Food Tests could be as a screen for EoE. Non-EoE subjects with falsely positive EoE Milk Tests had all been referred for endoscopies due to lower GI symptoms and went on to be diagnosed with IBS or prescribed IBS medications, suggesting food-specific T cells may contribute to IBS pathology. Studies specifically examining the performance of these functional T-cell assays in IBS cohorts are needed to interpret this finding, but despite inclusion of subjects with IBS as controls, the EoE Milk Test maintains a relatively high NPV of 92%.

We performed several evaluations to provide additional rigor to our EoE Milk Test. We found that the test performed similarly in subjects regardless of the degree of EoE activity and was independent of milk consumption. Further, and as expected, we observed that the magnitude of specific responses to TT gradually declined with time since vaccination. However, we did not observe an association between age or time since the last tetanus vaccination with any of our assay outcomes. Finally, the EoE Milk Test showed a high degree of repeatability, although most subjects in the repeatability cohort presumably did not have EoE diagnoses or milk allergy. Despite these important evaluations, additional data (e.g., reproducibility, comparison against a validation cohort) will be required prior to making the EoE Milk Test clinically available.

This study is limited primarily by its non-interventional, single-center design and by the composition of the enrolled cohorts. Because participants in the prospective study were recruited before their initial endoscopy results, the cohort was skewed toward controls without EoE, a bias we mitigated through statistical pruning. Additionally, participants with EoE pursued heterogeneous treatment courses. Food allergy status assignment was often precluded by the co-occurrence of diet and medication changes and by diet changes involving several foods at once. This design likely underrepresented patients with multiple contributing food allergies, and the resulting performance data should therefore be viewed as preliminary until validated in a multi-institutional interventional trial.

Additional limitations include sample-related exclusions. Low peripheral blood yields (<3 mL) or poor PBMC viability accounted for all 58 “insufficient sample” and most of the 38 “technical” exclusions, underscoring the need for adequate sample volume in future applications. Minor assay constraints included the requirement for background proliferation <20% and prior tetanus vaccination. Although nearly all children are immunized, verification of vaccination date was sometimes unavailable, limiting analyses of time-since-vaccination effects to Pennsylvania residents. Incorporating an antigen/tetanus threshold that adjusts for waning vaccine responses—and defining the minimal positive control threshold using an unvaccinated reference cohort—may improve future assay calibration.

Finally, these functional T-cell assays identify food-reactive peripheral memory CD4^+^ T cells but do not distinguish between EoE-mediated and IgE-mediated food allergy, nor between EoE and healthy controls. Thus, while promising as a diagnostic adjunct, their clinical utility should be interpreted within the broader context of EoE’s established diagnostic framework.

In conclusion, the EoE Food Tests represent a potentially significant advancement in the diagnostic landscape of EoE. Future research should focus on validating these findings in larger, multi-center cohorts and optimizing the assay’s integration into routine clinical practice via interventional studies, as well as expanding the testing framework to include additional EoE-causal foods such as egg and wheat. By refining our understanding of T-cell-mediated food responses, we may ultimately move toward more precise and patient-centered strategies for managing EoE.

## Figures and Tables

**Table 1:** Summary of subject demographic characteristics by clinical cohort.

Cohort	Milk Tolerant		Milk Allergic	Soy Tolerant		Soy Allergic
**Diagnosis**	**Control**	**EoE**	**EoE**	**Control**	**EoE**	**EoE**
Number	22	7	15	6	8	10
	Total: 29			Total: 14		
Mean age	14	13	8.6	11.7	10.1	10
	Total: 13.8			Total: 10.8		
% Male	31.8	28.6	40	66.7	37.5	70
	Total: 31			Total: 50		
% White	77.3	71.4	80	66.7	75	70
	Total: 75.9			Total: 71.4		
% NH/L	86.4	71.4	100	83.3	75	100
	Total: 82.8			Total: 78.6		
% Atopic	54.5	85.7	66.7	66.7	87.5	100
	Total: 62.1			Total: 78.6		

Abbreviations: NH/L = non-Hispanic/Latino. Combined control and EoE subjects in blue. Mean age in years.

## Data Availability

All primary data will be made available by the PI at reasonable request.

## Abbreviations

The following abbreviations are used in this manuscript:

EoE	Eosinophilic esophagitis
CHOP	Children’s Hospital of Philadelphia
EMR	Electronic Medical Record
PBMCs	Peripheral Blood Mononuclear Cells
CFSE	Carboxyfluorescein succinimidyl ester
ROC	Receiver Operating Characteristic
PPV	Positive predictive value
NPV	Negative predictive value
TP	True positive
TN	True negative
FP	False positive
FN	False negative
Eos/hpf	Eosinophils per high-power field

## Appendix A

**Table A1:** Summary of prospectively enrolled subjects’ demographic characteristics in Figure 1 and Figure 2.

Cohort	Milk Tolerant		Milk Allergic	Soy Tolerant		Soy Allergic
**Diagnosis**	**Control**	**EoE**	**EoE**	**Control**	**EoE**	**EoE**
Number	22	7	15	1	7	1
	Total: 29			Total: 8		
Mean age	14	13	8.6	5.4	9.6	11.4
	Total: 13.8			Total: 9.1		
% Male	31.8	28.6	40	100	28.6	0
	Total: 31			Total: 37.5		
% White	77.3	71.4	80	100	71.4	0
	Total: 75.9			Total: 75		
% NH/L	86.4	71.4	100	0	100	100
	Total: 82.8			Total: 87.5		
% Atopic	54.5	85.7	66.7	100	85.7	100
	Total: 62.1			Total: 87.5		

Abbreviations: NH/L = non-Hispanic/Latino. Combined control and EoE subjects in blue. Mean age in years.

**Table A2:** Summary of retrospectively enrolled subjects’ demographic characteristics in Figure 1 and Figure 7.

Cohort	Soy Tolerant		Soy Allergic
**Diagnosis**	**Control**	**EoE**	**EoE**
Number	5	1	9
	Total: 6		
Mean age	12.9	12	9.8
	Total: 12.8		
% Male	60	100	66.7
	Total: 66.7		
% White	60	100	77.8
	Total: 66.7		
% NH/L	100	100	100
	Total: 100		
% Atopic	60	100	100
	Total: 66.7		

Abbreviations: NH/L = non-Hispanic/Latino. Combined control and EoE subjects in blue. Mean age in years.

**Table A3:** Detailed demographic and clinical characteristics of the prospective study subjects included in figures.

ID	Cohort	Figures	Atopic Diagnoses	Race	Ethnicity	Sex	Age (Years)
1	Control (milk test)	Table 1 and Figure 1, Figure 2, Figure 5	None	W	NH/L	F	10
2	Control (milk test)	Table 1 and Figure 1, Figure 2, Figure 5	AR	O	NH/L	M	7
3	Control (milk test)	Table 1 and Figure 1, Figure 2, Figure 5	None	W	NH/L	M	6
4	Control (milk test)	Table 1 and Figure 1, Figure 2, Figure 5	AR, IgE-FA	W	NH/L	F	16
5	Control (milk test)	Table 1 and Figure 1, Figure 2, Figure 5	AR, AD, AS	W	NH/L	F	16
6	Control (milk test)	Table 1 and Figure 1, Figure 2, Figure 5	AR	W	NH/L	F	17
7	Control (milk test)	Table 1 and Figure 1, Figure 2, Figure 5	AR	O	NH/L	F	14
8	Control (milk test)	Table 1 and Figure 1, Figure 2, Figure 5	None	W	NH/L	F	16
9	Control (milk test)	Table 1 and Figure 1, Figure 2, Figure 5	None	W	NH/L	F	10
10	Control (milk test)	Table 1 and Figure 1, Figure 2, Figure 5	AR, AS, IgE-FA	MR	HL	F	17
11	Control (milk test)	Table 1 and Figure 1, Figure 2, Figure 5	None	W	NH/L	M	8
12	Control (milk test)	Table 1 and Figure 1, Figure 2, Figure 5	None	W	NH/L	F	15
13	Control (milk test)	Table 1 and Figure 1, Figure 2	AD	W	NH/L	F	15
14	Control (milk test)	Table 1 and Figure 1, Figure 2	AD, AR, AS	W	NH/L	F	12
15	Control (milk test)	Table 1 and Figure 1, Figure 2	None	W	NH/L	F	19
16	Control (milk test)	Table 1 and Figure 1, Figure 2	AR, AD, AS	W	NH/L	F	19
17	Control (milk test)	Table 1 and Figure 1, Figure 2	AR, AD, AS	B	NH/L	M	16
18	Control (milk test)	Table 1 and Figure 1, Figure 2	AR, IgE-FA	W	NH/L	M	18
19	Control (milk test)	Table 1 and Figure 1, Figure 2	None	W	HL	M	10
20	Control (milk test)	Table 1 and Figure 1, Figure 2	None	W	NH/L	F	12
21	Control (milk test)	Table 1 and Figure 1, Figure 2	AR, AS	B	NH/L	M	18
22	Control (milk test)	Table 1 and Figure 1, Figure 2	None	W	HL	F	17
23	Control (milk test)	Figure 1 and Figure 5	None	W	ND	M	16
24	Control (milk test)	Figure 1 and Figure 5	None	W	NH/L	F	14
25	Control (milk test)	Figure 1 and Figure 5	None	W	NH/L	M	14
26	Control (milk test)	Figure 1 and Figure 5	None	B	NH/L	M	13
27	Control (milk test)	Figure 1 and Figure 5	None	W	NH/L	M	12
28	Control (milk test)	Figure 1 and Figure 5	AR, AS	W	NH/L	M	11
29	Control (milk test)	Figure 1 and Figure 5	None	O	NH/L	M	10
30	Control (milk test)	Figure 1 and Figure 5	None	W	NH/L	F	10
31	Control (milk test)	Figure 1 and Figure 5	None	W	NH/L	M	8
32	Control (milk test)	Figure 1 and Figure 5	AS	W	NH/L	M	7
33	Control (milk test)	Figure 1 and Figure 5	AR	W	NH/L	F	7
34	Control (milk test)	Figure 1 and Figure 5	None	W	NH/L	F	6
35	Control (milk test)	Figure 1 and Figure 5	None	W	NH/L	F	5
36	Control (soy test)	Table 1 and Figure 1, Figure 7	AR	W	O	M	5
37	EoE to milk	Table 1 and Figure 1, Figure 2, Figure 3, Figure 4, Figure 5	AR, AD, AS, EoE	W	NH/L	M	13
38	EoE to milk	Table 1 and Figure 1, Figure 2, Figure 3, Figure 4, Figure 5	AR, AD, AS, IgE-FA, EoE	O	NH/L	F	6
39	EoE to milk	Table 1 and Figure 1, Figure 2, Figure 3, Figure 4, Figure 5	AR, AS, IgE-FA, EoE	W	NH/L	F	8
40	EoE to milk	Table 1 and Figure 1, Figure 2, Figure 3, Figure 4	AR, IgE-FA, EoE	W	NH/L	M	9
41	EoE to milk	Table 1 and Figure 1, Figure 2, Figure 3, Figure 4	AD, AR, IgE-FA, EoE	W	NH/L	F	4
42	EoE to milk	Table 1 and Figure 1, Figure 2, Figure 3, Figure 4	EoE	W	NH/L	M	13
43	EoE to milk	Table 1 and Figure 1, Figure 2, Figure 3, Figure 4	AD, AR, IgE-FA, EoE	O	NH/L	M	9
44	EoE to milk	Table 1 and Figure 1, Figure 2, Figure 3, Figure 4	EoE	W	NH/L	F	11
45	EoE to milk	Table 1 and Figure 1, Figure 2, Figure 3, Figure 4	AS, IgE-FA, EoE	W	NH/L	F	9
46	EoE to milk	Table 1 and Figure 1, Figure 2, Figure 3, Figure 4	EoE	W	NH/L	F	9
47	EoE to milk	Table 1 and Figure 1, Figure 2, Figure 3, Figure 4	EoE	W	NH/L	M	9
48	EoE to milk	Figure 1 and Figure 5	AR, EoE	W	NH/L	M	11
49	EoE to milk and soy	Table 1 and Figure 1, Figure 2, Figure 3, Figure 4, Figure 5, Figure 7	AR, AS, EoE	O	NH/L	F	11
50	EoE to milk. Soy tolerant	Table 1 and Figure 1, Figure 2, Figure 3, Figure 4, Figure 5, Figure 7	AR, AD, EoE	W	NH/L	M	9
51	EoE to milk. Soy tolerant	Table 1 and Figure 1, Figure 2, Figure 3, Figure 4, Figure 5, Figure 7	AR, AD, EoE	W	NH/L	F	3
52	EoE to milk. Soy tolerant	Table 1 and Figure 1, Figure 2, Figure 3, Figure 4, Figure 7	EoE	W	NH/L	F	6
53	EoE, milk and soy tolerant	Table 1 and Figure 1, Figure 2, Figure 5, Figure 7	AR, AD, AS, IgE-FA, EoE-OG	W	NH/L	F	16
54	EoE, milk and soy tolerant	Table 1 and Figure 1, Figure 2, Figure 3, Figure 5, Figure 7	AD, IgE-FA, EoE	B	HL	F	10
55	EoE, milk and soy tolerant	Table 1 and Figure 1, Figure 2, Figure 3, Figure 5, Figure 7	AS, EoE-OG	MR	HL	F	16
56	EoE, milk tolerant	Table 1 and Figure 1, Figure 2, Figure 3, Figure 5	IgE-FA, EoE	W	NH/L	F	17
57	EoE, milk tolerant	Table 1 and Figure 1, Figure 2, Figure 3, Figure 5	AD, AR, EoE-OG	W	NH/L	M	17
58	EoE, milk tolerant	Table 1 and Figure 1, Figure 2, Figure 3	EoE	W	NH/L	M	4
59	EoE, milk tolerant	Table 1 and Figure 1, Figure 2	AS, IgE-FA, EoE-OG	W	NH/L	F	15
60	EoE, milk tolerant	Figure 1 and Figure 5	IgE-FA, EoE	W	NH/L	F	18
61	EoE, soy tolerant	Table 1 and Figure 1, Figure 3, Figure 4, Figure 5, Figure 7	IgE-FA, EoE	W	NH/L	M	7
62	EoE, unknown	Figure 1 and Figure 5	AR, AS, EoE	W	NH/L	M	15
63	EoE, unknown	Figure 1 and Figure 5	AS, EoE	W	NH/L	M	9
64	EoE, unknown	Figure 1 and Figure 5	EoE	W	HL	M	6
65	EoE, unknown	Figure 1 and Figure 5	AD, AR, AS, IgE-FA, EoE	O	NH/L	M	3
66	EoE, unknown	Figure 1 and Figure 4	AR, EoE	W	NH/L	M	13
67	EoE, unknown	Figure 1 and Figure 4	EoE	W	NH/L	F	3
68	EoE, unknown	Figure 1, Figure 3 and Figure 6	AR, EoE	W	NH/L	M	17
69	EoE, unknown	Figure 1, Figure 3, Figure 5 and Figure 6	AD, AR, IgE-FA, EoE	W	NH/L	M	3
70	EoE, unknown	Figure 1, Figure 3 and Figure 5	EoE	W	NH/L	M	16
71	EoE, unknown	Figure 1, Figure 3 and Figure 5	AS, AR, EoE	W	NH/L	M	13
72	EoE, unknown	Figure 1, Figure 3 and Figure 5	IgE-FA, EoE	W	NH/L	M	17
73	EoE, unknown	Figure 1, Figure 3 and Figure 5	AS, AR, IgE-FA, EoE	O	NH/L	M	16
74	EoE, unknown	Figure 1, Figure 3 and Figure 5	EoE	O	NH/L	M	11
75	EoE, unknown	Figure 1, Figure 3 and Figure 5	AD, AR, AS, EoE	W	NH/L	M	11
76	EoE, unknown	Figure 1, Figure 3 and Figure 5	AR, AS, EoE	W	NH/L	M	17
77	EoE, unknown	Figure 1, Figure 3 and Figure 5	AR, AS, EoE	W	NH/L	M	10
78	EoE, unknown	Figure 1, Figure 3 and Figure 5	AD, AR, IgE-FA, EoE	W	NH/L	M	3
79	EoE, unknown	Figure 1, Figure 3 and Figure 5	AR, OAS, EoE	W	NH/L	M	13
80	EoE, unknown	Figure 1, Figure 3 and Figure 5	EoE	W	NH/L	M	11
81	EoE, unknown	Figure 1, Figure 3 and Figure 5	EoE	O	HL	M	7
82	EoE, unknown	Figure 1, Figure 3 and Figure 5	AD, AR, AS, IgE-FA, EoE	W	NH/L	M	8
83	EoE, unknown	Figure 1, Figure 3 and Figure 5	AR, AS, IgE-FA, EoE	MR	NH/L	F	7
84	EoE, unknown	Figure 1, Figure 3 and Figure 5	AS, EoE	W	NH/L	M	7
85	EoE, unknown	Figure 1, Figure 3 and Figure 5	EoE	B	NH/L	M	18
86	EoE, unknown	Figure 1, Figure 3 and Figure 5	AD, AR, IgE-FA, EoE	W	NH/L	M	17
87	EoE, unknown	Figure 1, Figure 3 and Figure 5	EoE	W	HL	F	18
88	EoE, unknown	Figure 1, Figure 3 and Figure 5	IgE-FA, EoE	MR	NH/L	M	5
89	EoE, unknown	Figure 1, Figure 3 and Figure 5	EoE	W	NH/L	F	10
90	EoE, unknown	Figure 1, Figure 3 and Figure 5	AS, AR, IgE-FA, EoE	W	NH/L	M	12
91	EoE, unknown	Figure 1, Figure 3 and Figure 5	AS, AR, EoE	O	HL	M	18
92	EoE, unknown	Figure 1, Figure 3 and Figure 5	IgE-FA, EoE	W	NH/L	F	14
93	EoE, unknown	Figure 1, Figure 3, Figure 4 and Figure 5	AR, IgE-FA, EoE	W	NH/L	M	11
94	EoE, unknown	Figure 1, Figure 3, Figure 4 and Figure 5	AR, AD, IgE-FA, EoE	B	NH/L	F	17
95	EoE, unknown	Figure 1, Figure 3 and Figure 4	IgE-FA, EoE	W	NH/L	M	12
96	EoE, unknown	Figure 1, Figure 3 and Figure 4	AR, AD, EoE	O	NH/L	M	5
97	EoE, unknown	Figure 1, Figure 3 and Figure 4	AR, EoE	W	NH/L	M	4
98	EoE, unknown	Figure 1, Figure 3 and Figure 4	AR, AD, IgE-FA, EoE	B	NH/L	M	7
99	EoE, unknown	Figure 1 and Figure 3	IgE-FA, EoE	W	NH/L	M	16
100	EoE, unknown	Figure 1 and Figure 3	OAS, EoE	W	NH/L	F	11
101	EoE, unknown	Figure 1 and Figure 3	EoE	W	NH/L	M	7
102	EoE, unknown	Figure 1 and Figure 3	AS, AR, EoE	W	NH/L	M	12
103	EoE, unknown	Figure 1 and Figure 3	AS, AR, IgE-FA, EoE	W	NH/L	F	16
104	EoE, unknown	Figure 1 and Figure 3	AD, EoE	W	NH/L	F	2
105	EoE, unknown	Figure 1 and Figure 3	EoE	W	NH/L	M	12
106	EoE, unknown	Figure 1 and Figure 3	EoE	W	NH/L	F	8
107	EoE, unknown	Figure 1 and Figure 3	AR, AS, IgE-FA, EoE	W	NH/L	F	16
108	EoE, unknown	Figure 1 and Figure 3	AD, IgE-FA, EoE	W	NH/L	M	5
109	EoE, unknown	Figure 1 and Figure 3	OAS, EoE	O	HL	M	13
110	EoE, unknown	Figure 1 and Figure 3	EoE	W	NH/L	M	13
111	EoE, unknown	Figure 1 and Figure 3	AR, EoE	W	NH/L	M	14
112	EoE, unknown	Figure 1 and Figure 3	AR, AS, OAS, EoE	W	NH/L	M	19
113	EoE, unknown	Figure 1 and Figure 3	AD, AR, IgE-FA, EoE	MR	HL	M	4
114	EoE, unknown	Figure 1 and Figure 3	EoE	O	NH/L	M	17
115	EoE, unknown	Figure 1 and Figure 3	AS, IgE-FA, EoE	B	NH/L	M	3

Abbreviations: AD = atopic dermatitis, AR = allergic rhinitis, AS = asthma, B = Black, EoE = eosinophilic esophagitis, EoE-OG = EoE-outgrown, F = Female, HL = Hispanic/Latino, IgE-FA = IgE-mediated food allergy, M = male, MR = multiracial, ND = No data, NH/L = non-Hispanic/Latino, OAS = oral allergy syndrome, O = other, W = White.

**Table A4:** Detailed demographic and clinical characteristics of the retrospective study subjects included in figures.

ID	Cohort	Figures	Atopic Diagnose(s)	Race	Ethnicity	Sex	Age (Years)
1	Control (soy test)	Table 1 and Figure 1, Figure 7	None	W	NH/L	M	17
2	Control (soy test)	Table 1 and Figure 1, Figure 5, Figure 7	None	W	NH/L	M	15
3	Control (soy test)	Table 1 and Figure 1, Figure 5, Figure 7	AR, OAS	W	NH/L	M	15
4	Control (soy test)	Table 1 and Figure 1, Figure 7	AD, IgE-FA	A	NH/L	F	2
5	Control (soy test)	Table 1 and Figure 1, Figure 7	AR	B	NH/L	F	15
6	EoE, soy tolerant	Table 1 and Figure 1, Figure 5, Figure 7	AS, EoE	W	NH/L	M	12
7	EoE to soy	Table 1 and Figure 1, Figure 7	AD, AS, IgE-FA, EoE	W	NH/L	F	11
8	EoE to soy	Table 1 and Figure 1, Figure 7	AS, IgE-FA, EoE	W	NH/L	F	17
9	EoE to soy	Table 1 and Figure 1, Figure 7	AS, EoE	W	NH/L	M	16
10	EoE to soy	Table 1 and Figure 1, Figure 7	IgE-FA, EoE	O	NH/L	M	5
11	EoE to soy	Table 1 and Figure 1, Figure 7	AR, AS, IgE-FA, EoE	W	NH/L	M	10
12	EoE to soy	Table 1 and Figure 1, Figure 7	IgE-FA, EoE	W	NH/L	M	14
13	EoE to soy	Table 1 and Figure 1, Figure 7	AR, AS, IgE-FA, EoE	O	NH/L	M	8
14	EoE to soy	Table 1 and Figure 1, Figure 7	IgE-FA, EoE	W	NH/L	F	6
15	EoE to soy	Table 1 and Figure 1, Figure 7	AR, IgE-FA, EoE	W	NH/L	M	3
16	Control (milk test)	Figure 5	None	W	NH/L	F	17
17	Control (milk test)	Figure 5	None	W	NH/L	M	17
18	Control (milk test)	Figure 5	None	W	NH/L	M	16
19	EoE, unknown	Figure 5	AS, EoE	W	NH/L	M	16
20	Control (milk test)	Figure 5	OAS	W	NH/L	F	16
21	EoE, unknown	Figure 5	EoE	W	NH/L	M	15
22	EoE, milk tolerant	Figure 5	AR, AS, IgE-FA, EoE	W	NH/L	M	14
23	EoE to milk	Figure 5	AD, AR, AS, EoE	O	HL	M	13
24	Control (milk test)	Figure 5	None	W	NH/L	M	12
25	EoE, unknown	Figure 5	EoE	W	NH/L	F	12
26	Control (milk test)	Figure 5	None	MR	HL	F	11
27	EoE to milk	Figure 5	IgE-FA, EoE	W	NH/L	F	10
28	EoE to milk	Figure 5	AR, AS, EoE	W	NH/L	F	11
29	Control (milk test)	Figure 5	None	W	NH/L	M	9
30	EoE to milk	Figure 5	AR, AD, IgE-FA, EoE	W	NH/L	M	9
31	EoE to milk	Figure 5	EoE	O	NH/L	M	6
32	Control (milk test)	Figure 5	None	W	NH/L	M	5
33	EoE to milk	Figure 5	AD, AS, IgE-FA, EoE	O	NH/L	F	4
34	EoE, unknown	Figure 5	EoE	W	NH/L	M	3
35	Control (milk test)	Figure 5	None	O	NH/L	F	17
36	EoE, unknown	Figure 5	EoE	W	NH/L	F	17
37	EoE to milk	Figure 5	AR, EoE	W	NH/L	F	18
38	Control (milk test)	Figure 6 and Figure 7	None	W	NH/L	M	18
39	EoE, milk tolerant	Figure 5	AS, EoE	W	NH/L	F	18
40	Control (milk test)	Figure 5	None	W	NH/L	F	18
41	EoE to milk	Figure 6	EoE	MR	NH/L	M	9
42	Control (milk test)	Figure 6	None	W	NH/L	F	12
43–49	Control (milk test)	Figure 6	NA	NA	NA	NA	NA

Abbreviations: A = Asian, AD = atopic dermatitis, AR = allergic rhinitis, AS = asthma, EoE = eosinophilic esophagitis, F = female, HL = Hispanic/Latino, IgE-FA = IgE-mediated food allergy, M = male, MR = multiracial, NA = not available, NH/L = non-Hispanic/Latino, OAS = oral allergy syndrome, O = other, W = White.
